# Injection laryngoplasty of human adipose-derived stem cell spheroids with hyaluronic acid-based hydrogel improves the morphological and functional characteristics of geriatric larynx

**DOI:** 10.1186/s40824-022-00261-x

**Published:** 2022-04-05

**Authors:** Doh Young Lee, Young Hwan Choi, Ji Suk Choi, Min Rye Eom, Seong Keun Kwon

**Affiliations:** 1 Department of Otorhinolaryngology-Head and Neck, Seoul National University Boramae Medical Center, Seoul National University College of Medicine, Seoul, Republic of Korea; 2 Translational Tissue Engineering Center and Wilmer Eye Institute, Johns Hopkins University, Baltimore, MD 21287 USA; 3 Bio-MAX/N-Bio Institute, Institute of Bioengineering, Seoul National University, Seoul, Republic of Korea; 4 Department of Otorhinolaryngology-Head and Neck Surgery, Biomedical Research Institute, Seoul National University Hospital, Seoul, Republic of Korea; 5 Sensory Organ Research Institute, Medical Research Center, Seoul National University, Seoul, Republic of Korea; 6 Department of Otorhinolaryngology-Head and Neck Surgery, Seoul National University Hospital, 101, Daehak-ro, Jongno-gu, Seoul, 03080 Republic of Korea; 7 Cancer Research Institute, Seoul National University, Seoul, Republic of Korea

**Keywords:** Geriatric larynx, Stem cell, Spheroids, Presbylaryngis, Injection laryngoplasty

## Abstract

**Aim:**

As the geriatric population increased, the need of treatment for laryngeal atrophy and dysfunction increased. This study was performed to evaluate the effects of injection of human adipose-derived stem cell (hASC) spheroid-loaded catechol-conjugated hyaluronic acid (HA-CA) hydrogel on therapeutic rejuvenation of the geriatric larynx.

**Methods:**

Stem cell spheroids with hyaluronic acid-based hydrogel were injected into the laryngeal muscles of 18-month-old Sprague–Dawley rats. The effects of hASC spheroids were examined in the following four groups: SHAM, injected with PBS; GEL, injected with HA-CA hydrogel; MONO, injected with single hASCs in HA-CA hydrogel; and SP, injected with hASCs spheroids in HA-CA hydrogel. The rejuvenation efficacy in geriatric laryngeal muscle tissues at 12 weeks postinjection was evaluated and compared by histology, immunofluorescence staining, and functionality analysis.

**Results:**

Total myofiber cross-sectional area and myofiber number/density, evaluated by detection of myosin heavy chain with antibodies against laminin and fast myosin heavy chain, were significantly higher in the SP group than in the other groups. The lamina propria of the larynx was evaluated by alcian blue staining, which showed that the HA was increased significantly in the SP group compared to the other groups. In functional analysis, the glottal gap area was significantly reduced in the SP group compared to the other groups. The phase difference in the vocal fold during vibration was also smaller in the SP group than in the other groups, but the difference did not reach statistical significance.

**Conclusion:**

Injection of hASC spheroids with hyaluronic acid-based hydrogel improves the morphological and functional characteristics of geriatric larynx.

**Graphical abstract:**

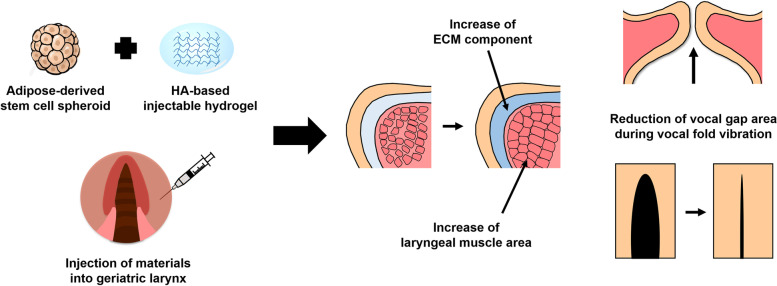

**Supplementary Information:**

The online version contains supplementary material available at 10.1186/s40824-022-00261-x.

## Introduction

The rates of voice-related problems are increasing worldwide with the aging of the population, and they are among the most common and undertreated problems in the geriatric population [1]. About 20–30% of elderly people are thought to have vocal problems, for which only about 15% seek medical attention [2, 3]. Among the various causes of geriatric dysphonia, atrophy of the vocal fold (presbylaryngis) has increased rapidly and is attracting considerable attention. The pathophysiology of atrophy involves atrophy and hypotonicity of the vocalis muscles, epithelial atrophy with increased collagen and decreased nonfibrous protein of the extracellular matrix, and deterioration of the framework structure (cartilage and joints) [4, 5].

Typical symptoms of presbylaryngis are mild/moderate dysphonia, lack of volume/projection, and vocal fatigue, all of which have a significantly negative impact on the quality of life and ability to participate in society [6]. As presbylaryngis is a form of glottic insufficiency, it can be treated with methods to increase glottic contact, such as voice therapy, injection laryngoplasty, and medialization thyroplasty [7–9]. In addition to medialization by injection of space-occupying materials (e.g., calcium hydroxyapatite), the use of various substances, such as growth factors, platelet-rich plasma, and stem cells, has been examined to restore the sophisticated and unique structure of vocal-fold mucosa and muscles both in vivo and in clinical trials [10–12]. Regeneration of laryngeal tissues and restoration of intrinsic laryngeal functions are challenging problems that have yet to be addressed by existing injectable materials. Previously, with experience of injection laryngoplasty of diverse materials, including polycaprolactone, Pluronic F127, poly (lactic-co-glycolic acid) (PLGA), and adipose-derived extracellular matrix (ECM) [13–18], we have accumulated various experimental and analytical methods to assess the properties of injectable materials.

HA is an essential component of ECM and is made up of repeating units of disaccharides that can be easily modified with different crosslinkable functional groups. Among them, HA-CA hydrogel has been widely explored and used as a highly biocompatible, injectable, adhesive and in-situ forming hydrogel for therapeutic cell delivery [19–23] Mesenchymal stem cells (MSCs) show site-specific differentiation and can improve the mechanical properties of connective tissue [24]. Our previous studies showed that MSCs are potentially beneficial in tracheal regeneration and oral mucosal ulcer healing [25–27]. While the use of MSCs has also been studied for vocal-fold atrophy [28], spheroids of MSCs and mesenchymal stroma have not been studied despite their enhanced multipotency in vitro [29]. In the present study, we injected hASC spheroid-loaded hyaluronic-acid (HA)-based hydrogel into the geriatric larynx to evaluate its effects on therapeutic rejuvenation.

## Materials and methods

### Materials

HA (200 kDa; Lifecore Biomedical, Chaska, MN, USA), N-hydroxysulfosuccinimide (sulfo-NHS-sodium; Sigma-Aldrich, St. Louis, MO, USA), N-(3-dimethylaminoprophy)-N′-ethylcarbodiimide (EDC hydrochloride; Thermo Fisher Scientific, Wilmington, DE, USA), 3,4-dihydroxyphenethylamine hydrochloride (dopamine hydrochloride; Sigma-Aldrich), dialysis membrane (Cellu Sep T2, MW cut-off 6–8 kDa; Membrane Filtration Products Inc., Seguin, TX, USA), 0.1 M hydrochloric acid solution (0.1 M HCL; Daejung, Busan, Republic of Korea), sodium periodate (NaIO_4_; Sigma-Aldrich), and sodium hydroxide (NaOH; Sigma-Aldrich) were purchased from the sources shown.

### Adipose-derived stem cell culture

Human adipose-derived stem cells (hASCs; STEMPRO® Human Adipose-Derived Stem Cells; Invitrogen, Carlsbad, CA, USA) were used in this experiment. The hASCs were cultured in Dulbecco’s Modified Eagle Medium (DMEM) supplemented with 10% FBS, 10 ng/mL epidermal growth factor, 10 ng/mL basic fibroblast growth factor, 100 U/mL penicillin, and 100 μg/mL streptomycin, and were used at passage 6–8 in the experiments.

### Preparation of hASCs spheroid

**Fig. 1:**
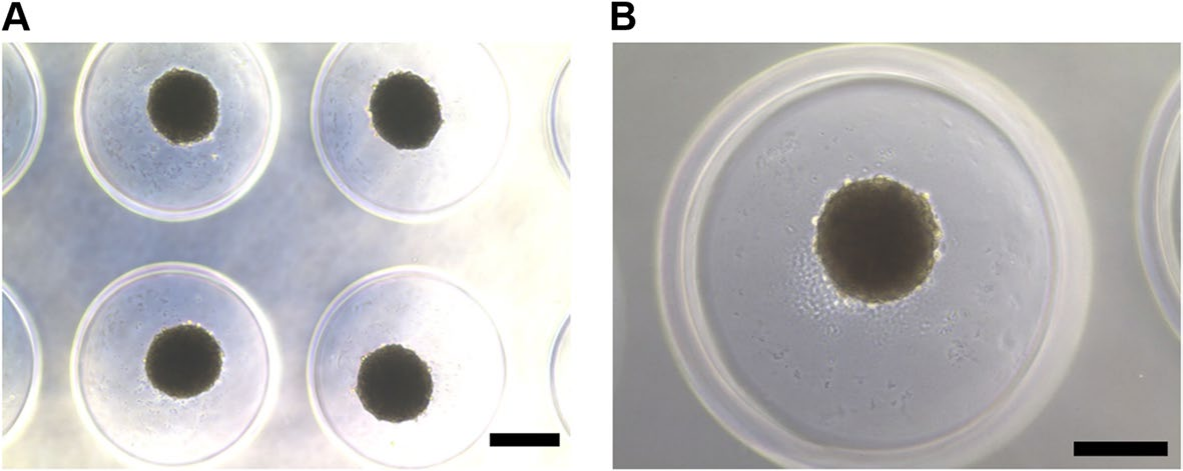
Formation of hASC-spheroids from agarose mold. A prepared agarose mold consisting of 35 cylindrical wells was placed in the wells of 24-well plates. Aliquots of 2.5 × 10^5^ hASCs were seeded and cultured in the molds. **A** Aliquots of 2.5 × 10^5^ hASCs were separated into 35 cylindrical wells of the agarose molds (scale bar: 200 μm), **B** and each cylindrical well-formed spheroids about 200 μm in diameter after 72 h of culture (scale bar: 200 μm)

To form hASC spheroids, commercial MicroTissues® 3D Petri Dish® micro-mold spheroids (Sigma-Aldrich) were used as molds in hydrogel-replicated microwells. The autoclaved molds were prepared and transferred into a hood. Sterilized 1.5% w/v agarose (UltraPure™ Agarose; Invitrogen) in phosphate buffered saline (PBS) was transferred into the hood and stirred with a magnetic bar on a hotplate. The agarose solution was pipetted into the molds and allowed to solidify at room temperature for 5 min. The agarose molds were transferred into individual wells of 24-well plates. The molds were then immersed in PBS and stored at 4 °C until use. The prepared hASCs were seeded on the mold and cultured with cell culture medium at a density of 2.5 × 10^5^ cells/mold in 24-well plates. After 72 h, 35 spheroids about 200 μm in diameter were formed from a mold (Fig. 1).

### Preparation of hASC spheroids-loaded HA hydrogel

In order to deliver hASC spheroids, an in situ-forming HA-CA hydrogel was used as a carrier. The HA-CA gel was prepared with the same method according to our previous studies (Supplementary Figs. 1, 2) [19, 20]. Briefly, HA-CA precursor solution was prepared at 3.33% (w/v) in PBS (pH 7.4) and a mixture of 4.5 mg/mL NaIO_4_ and 0.4 M NaOH was prepared as a crosslinker at a ratio of 100:1. To load hASC (2.5 × 10^5^ cell) or hASC spheroids (2.5 × 10^5^ cell, 35 spheroids) in the HA-CA hydrogel, 7 μL of hASC or hASC spheroids were added to 12 μL of the HA-CA precursor solution. Subsequently, 1 μL of the crosslinker were added into the mixture of HA-CA precursor solution and cells. After evenly mixing, the HA-CA hydrogel loaded hASC or hASC spheroids was loaded into a 25 μL of Hamilton syringe (luer tip 702LT) before injection.

### Injection of hASC-loaded HA hydrogel into geriatric rat laryngeal muscle

**Fig. 2:**
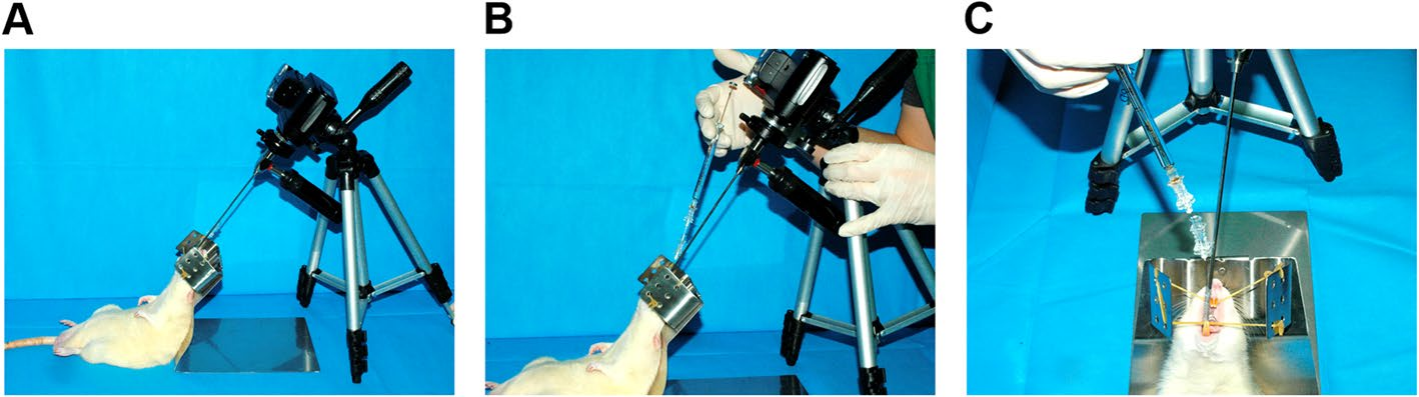
Injection of hASC spheroid-loaded HA-based hydrogel into geriatric rat laryngeal muscle using a laryngoscope. Under live imaging guidance, the hASC spheroid-loaded HA-based hydrogel was injected into the laryngeal muscle of geriatric rats. **A** Preparation of the injection laryngoscope equipped with a 25G spinal needle syringe containing hASC spheroid-loaded hydrogel. **B**, **C** Anesthetized aged SD rats were fixed using a custom-made frame and hASC spheroid-loaded hydrogel was injected into the laryngeal muscle using a stationary laryngoscope

All animal experiments were conducted using protocols approved by the Institutional Animal Care and Use Committee of Seoul National University (IACUC No, 16–0009-C2A0), Republic of Korea. Male Sprague–Dawley rats (SD rats, 18 months old) were purchased from Young Bio (Young Bio Inc., Gyeonggi-do, Republic of Korea). Rats were anesthetized by intraperitoneal injection of Zoletil 50 (tiletamine/zolazepam, 0.6 mL/kg) and Rompun (xylazine, 0.4 mL/kg). The preparation and assembly of the injection laryngoscope were described in our previous report (Fig. 2A) [17]. Briefly, a 25-gauge spinal needle mounted with Hamilton syringe (Hamilton syringe, luer tip 702LT, volume 25 μL) within a modified 20-gauge spinal needle (Tae Chang Industrial Co., Republic of Korea) with was attached to a 4.0 mm, 30° rigid endoscope (Richards, Knittlingen, Germany). The assembled endoscope with a Light-Emitting Diode (LED) based light source (Mediview, UMT-511, Australia) was mounted with a digital camera (Nikon Coolpix 4500, Japan). Accordingly, the prepared material was injected (each injection 20 μL/rat) into the right vocal fold of the rats (each group, *n* = 4) under live imaging guidance (Fig. 2B, C).

### Grouping according to the treatment

Experimental animals (18-month-old SD rats) were divided into four groups: sham, injected with PBS; GEL, injected with HA-CA hydrogel; MONO, injected with single hASCs in HA-CA hydrogel; and SP, injected with 35 hASCs spheroids in HA-CA hydrogel.

### Histological evaluation of laryngeal tissues

Twelve weeks post-injection, the larynges from each group (*n* = 4) were isolated and fixed with 4% paraformaldehyde solution (4% PFA; Bio Solution Co., Seoul, Republic of Korea). The samples were prepared for hematoxylin and eosin (H&E) staining, alcian blue (AB) staining, and immunofluorescence staining as described previously [17]. H&E- and AB-stained tissue sections were observed under an optical microscope. For immunofluorescence staining, tissue sections were immunostained with anti-laminin (ab11575) and anti-fast myosin skeletal heavy chain (MHC, ab51263) antibodies. After incubation, sections were washed and incubated with Alexa Fluor 488 goat anti-mouse or Alexa Fluor 594 goat anti-rabbit secondary antibody. The nuclei were counterstained with 4′,6-diamidino-2-phenylindole (DAPI). Histological sections were analyzed using Image J Software (NIH, Bethesda, MD, USA).

### Functional assessment of larynx using high-speed camera

**Fig. 3:**
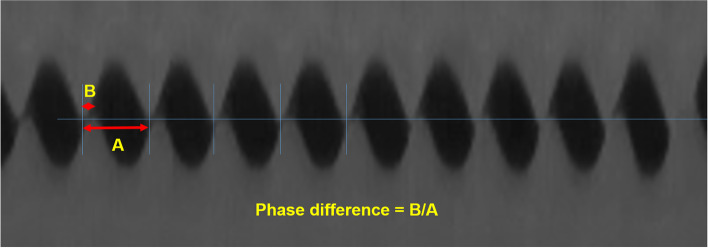
Measurement of phase difference in vocal-fold vibration. Phase difference was measured by comparing the cycles of bilateral vocal-fold vibration and defined as the ratio of the length of the phase discrepancy in one cycle (**B**) to the total length of one cycle (**A**)

To evaluate the functionality of the larynx, vocal-fold vibrations were examined using an excised laryngeal setup similar to our previous studies [15, 17]. To analyze the glottal gap area and phase difference in the vocal fold, the region of the vocal fold was specified with 28 horizontal 104 vertical pixels using Motion Studio™ software (version: 2.12.19; IDT Manufacturing, Pasadena, CA, USA). One cycle of vocal-fold vibration was composed of 10 serial images. Images with minimal glottal gap area were chosen from 10 serial images and the glottal gap area was measured using Image J software (four cycles of each sample were analyzed). Phase difference was measured by comparing the cycles of bilateral vocal-fold vibration and defined as the ratio of the length of phase discrepancy in one cycle to the total length of one cycle (Fig. 3).

### Statistical analysis

Data are presented as means ± standard deviation (SD). Continuous outcomes were analyzed using independent t-tests between groups of two, and one-way analysis of variance (ANOVA) with post-hoc test among groups of three or more. In all analyses, *P* < 0.05 was taken to indicate statistical significance.

## Results

### Histologic evaluation

**Fig. 4:**
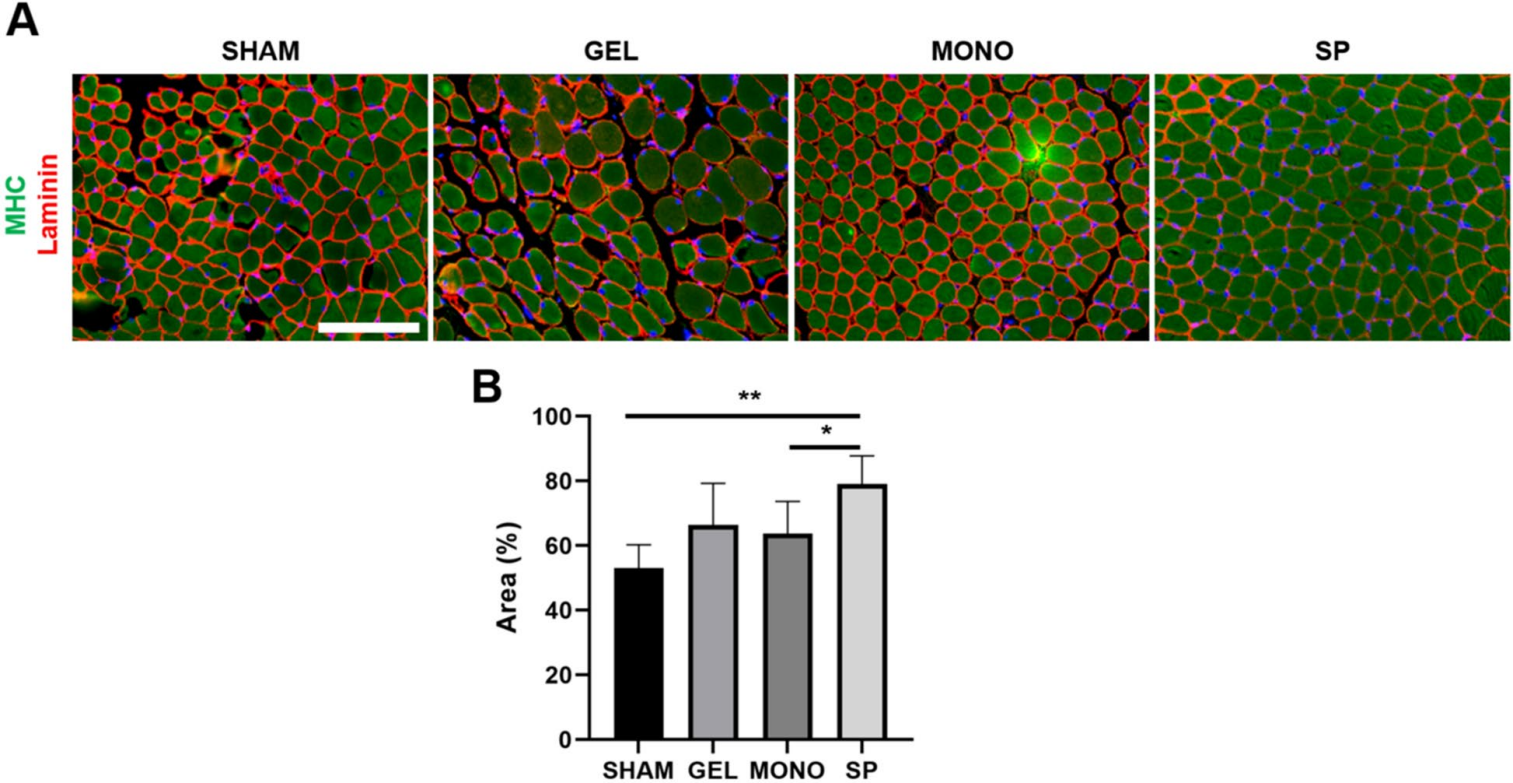
Effects of hASC spheroid-loaded HA-based hydrogel on hypertrophy of aged laryngeal muscle. **A** Representative images of laminin (red) and fast-myosin heavy chain (MHC, green) staining of each group (scale bar: 100 μm). **B** Total myofiber cross-sectional area was much greater in the SP group than in the other groups (*p < 0.05, **p < 0.01)

**Fig. 5:**
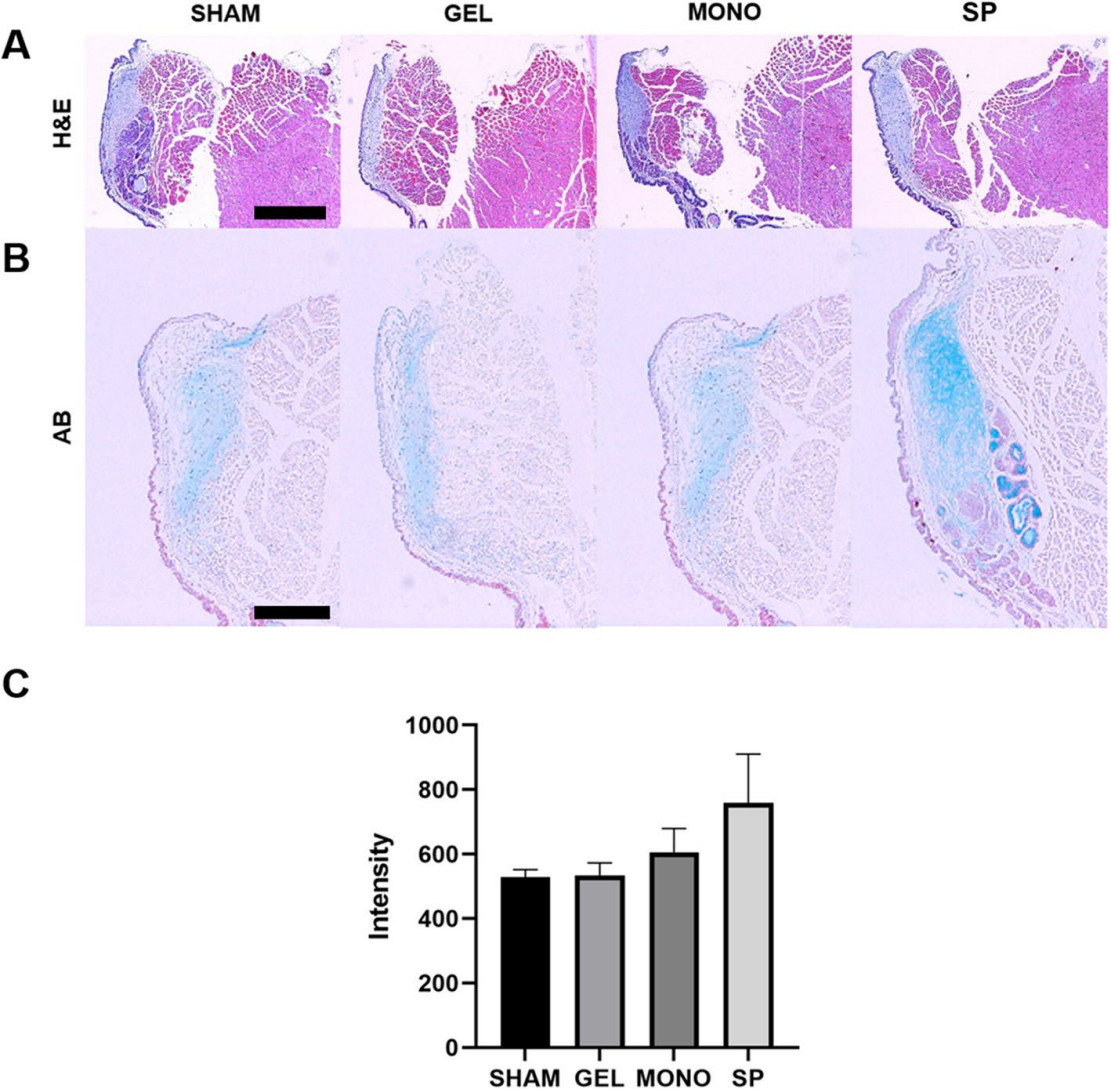
Effects of hASC spheroid-loaded HA-based hydrogel on the components of the lamina propria. Representative images of H&E (**A**) and alcian blue (**B**) staining of each group (scale bars: **A** 400 μm, **B** 200 μm). **C** The composition of hyaluronic acid in the lamina propria was significantly increased in the SP group compared to the other groups. The intensity of alcian blue staining was measured and compared, except in the area of submucosal glands, using Image J software. ANOVA revealed that four groups showed significant difference (*p* = 0.009), while independent t-test did not attain statistical significance of two group comparison. Scale bar of A = 400 μm, B = 200 μm

Morphological changes in laryngeal intrinsic muscles were analyzed by staining the fibrous connective tissue and myosin heavy chain with antibodies against laminin and fast myosin heavy chain, respectively (Fig. 4). Total myofiber cross-sectional area was much higher in the SP group than in the other groups (*p* = 0.003) (Fig. 4B). Consequently, the area between myofibers was reduced in the SP group compared to the other groups. The lamina propria of the larynx was evaluated by AB staining, which showed that the amount of HA was significantly increased in the SP group compared to the other groups (*p* = 0.009) (Fig. 5).

### Functional evaluation

**Fig. 6:**
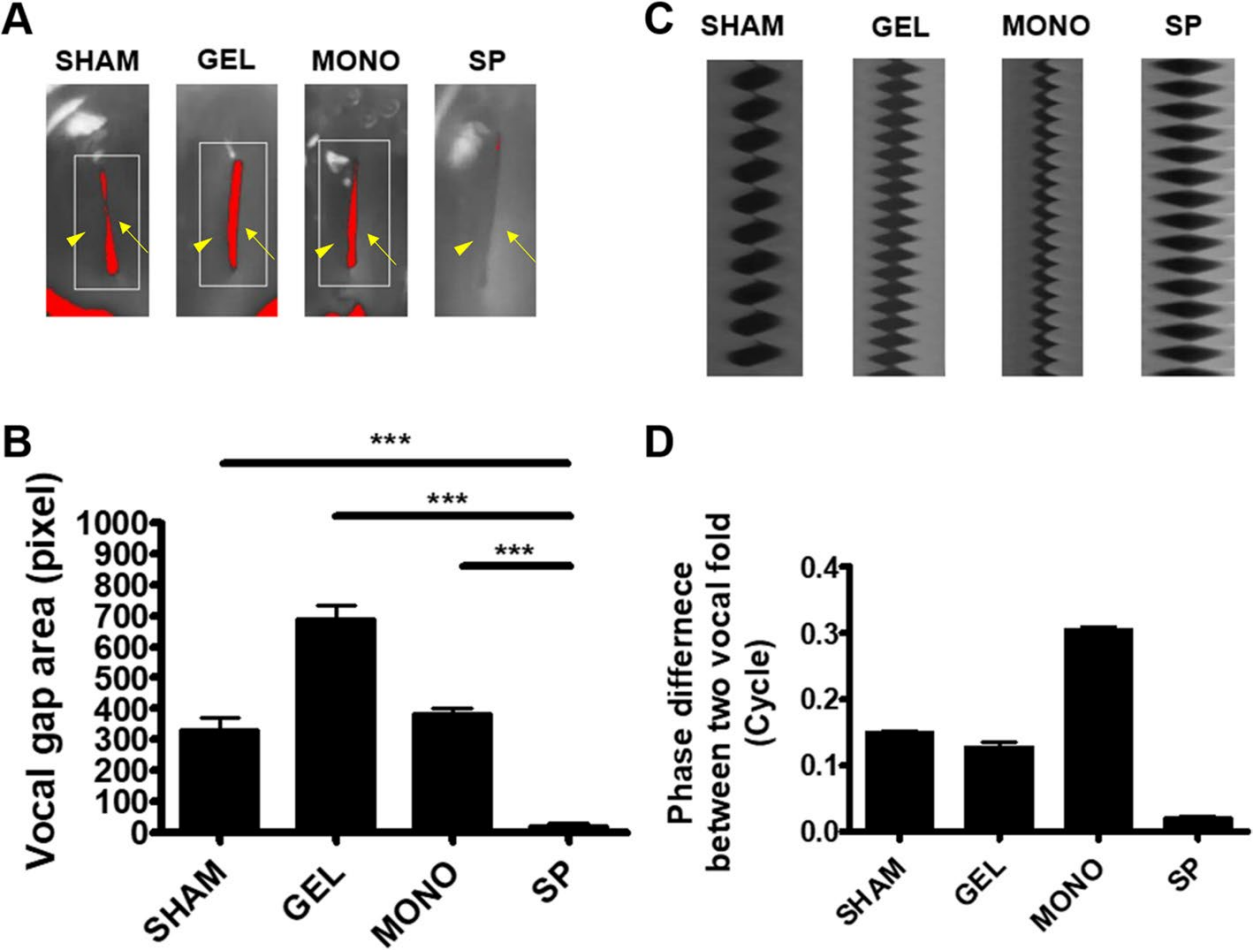
Functional analysis of the larynx. Twelve weeks post-injection, the rat larynges were isolated and analyzed using a high-speed camera setup. **A** Representative images of each minimal glottal gap (red-colored area) of the vocal fold (arrowhead, right vocal fold; arrow, left vocal fold). **B** The glottal gap area was significantly reduced in the SP group compared to the other groups. **C** Videokymographic analysis of vocal-fold motion. **D** The phase difference of the vocal fold during vibration was also smaller in the SP group than in the other groups, but the difference did not reach statistical significance (*p < 0.05, **p < 0.01, ***p < 0.005)

The ability to reduce the glottal gap area in the geriatric larynx was evaluated by analyzing vocal-fold vibration using an excised laryngeal setup (Fig. 6). The glottal gap area was significantly reduced in the SP group compared to the other groups (Fig. 6A and B). The phase difference in the vocal fold during vibration was also smaller in the SP group than in the other groups, but the difference did not reach statistical significance (Fig. 6C and D).

## Discussion

This study demonstrated that injection of hASC spheroids with HA-based hydrogel into the aged larynx improved the morphological characteristics in terms of increased intrinsic laryngeal muscles and HA in the lamina propria. Functional analysis showed that the phase difference in the mucosal wave was minimal with hASC spheroid injection, while other injections, including single hASCs, showed substantial phase differences in vibration cycles.

Three layers of intracartilaginous laryngeal structure undergo changes with age. Loss of muscle mass with age in the human thyroarytenoid muscle has been reported in addition to decreased innervation and metabolic activity of mitochondria [30]. The thickness of the epithelium usually decreases, and the lamina propria shows a variety of changes, including thickening/edema of the superficial layer and degeneration/atrophy of elastic fibers [31]. As dysfunction of the larynx due to aging can lead to problems with speech and social communication, several studies have been performed that used tissue-engineering techniques to address these issues. Many studies focused on restoration of volume at the area of muscle using various biomaterials [32]. In our previous study, we examined the use of injectable basic fibroblast growth factor (bFGF)-loaded alginate (ALG)/HA hydrogel for rejuvenation of geriatric laryngeal muscles, which showed increased expression of myogenic regulatory factor-related genes, hypertrophy of muscle fibers, proliferation of muscle satellite cells, and angiogenesis and decreased interstitial fibrosis [17].

As MSCs have self-renewal capability and tissue regeneration potential by differentiation and maturation into specific phenotypes, they have remarkable potential for clinical applications [33, 34]. The research library of the National Institutes of Health (NIH) includes reports of 993 clinical studies in which MSCs have been used to treat different diseases. The trials with MSCs frequently failed in controlled studies with large cohorts, probably because of lack of comprehension of the regenerative and immunomodulatory mechanisms. MSC spheroids showed considerable changes in gene expression pattern [35], and 3D cell culture has emerged as a new therapeutic alternative [36, 37]. There is evidence that MSC spheroids show better stemness, angiogenesis, differential potential, paracrine, and immunomodulatory effects than single MSCs [38–40].

In this study, we found that muscle volume increased to a significantly greater extent in the SP group than in the other groups, including the MONO group. In addition, the interstitial space decreased, although we did not evaluate whether this was due to decreased fibrosis or passive compression of the space by increased muscle volume. According to in vivo studies evaluating the therapeutic effects of hASC spheroids, wound healing and regeneration are possible with various cytokines and growth factors, including fibroblast growth factor, vascular endothelial growth factor, hepatocyte growth factor, etc., while in vitro studies usually evaluated stemness based on expression of the transcription factors Nanog, Oct-3/4, Sox-2, Klf4, c-Myc, and STAT3, in addition to surface markers, such as CD105, CD90, CD73, and CD34 [39, 41, 42]. As impaired muscle repair characterized by uncontrolled ECM remodeling results in the formation of fibrotic tissue in skeletal muscle [43], restoration of muscle volume may result in decreased interstitial fibrosis.

In addition, this study showed that the composition of HA in the lamina propria was increased significantly in the SP group compared to the other groups. HA, as one of the main components of the lamina propria in the larynx, plays a significant role in phonation via its vibratory potential. As most cases of presbylaryngis show decreased thickness of the epithelium and lamina propria and lack of vibratory tissue, increased HA in the lamina propria may result in improvements in the voice. Several studies have shown that the differentiation and activation of stem cells were facilitated by ECM of various organs, such as the kidney, liver, heart, and lung [44–46]. In terms of vocal-fold injury, Lungova et al. showed that applying human embryonic stem cells on gels composed of collagen and vocal-fold fibroblasts increased the vocal-fold lamina propria [47]. A study using bone-marrow-derived mesenchymal stromal cells revealed increased HA distribution and decreased dense collagen networks, resulting in improved biomechanical properties [48]. As spheroids of stem cells showed better regenerative potential, our study showed a greater increase in HA in the SP group than in the MONO group.

Most laryngeal injection procedures and injected materials can influence the mucosal wave. As vocal dysphonia is the main reason for treatment of geriatric larynx, impairment of the mucosal wave by injected material should be avoided because abnormalities in the mucosal wave are correlated with voice dysfunction, particularly hoarseness. Restoration of a normal mucosal wave is indicated by symmetric and periodic sinusoidal functions with sustained inter-vocal fold contact between cycles [49]. Symmetric mucosal wave was observed in the SP group, which showed the minimal phase difference in bilateral vocal fold vibration. In comparison to the sham group, the MONO group showed a negative impact on mucosal wave. As the injection itself and injection material induce inflammatory responses in the injected area, changes in the viscoelasticity of the overall laryngeal inner structure may have influenced the mucosal wave. As discussed above, hASC spheroids were superior to single cells in terms of immunomodulatory function and thereby may evoke minimal surrounding inflammation with a better mucosal wave.

Vocal-fold bowing and glottal gap are typical findings of presbylarynx. For a clear voice, glottal contact should be maintained during vocal-fold adduction. The SP group in this study had a much smaller vocal gap area compared to the other groups. As with the microscopy findings, appropriate muscle volume in the SP group may have resulted in a reduced vocal gap area.

## Conclusion

In this study, we showed that injection of hASC spheroids with a carrier enhanced the muscle volume of the geriatric larynx without a negative effect on the mucosal wave. Although the results did not include the characteristics of the young larynx, hASC spheroids were superior to single hASCs in terms of restoration of larynx muscle volume. In addition, although histological and functional changes beyond 12 weeks must be assessed to elucidate the effects of hASC spheroids on the more aged larynx, this study is important in that it is the first to use hASC spheroids in the treatment of geriatric larynx. Further studies involving analysis of the mechanism underlying the effects of hASC spheroids and with the loading of various cytokines and growth factors to improve the characteristics of lamina propria are needed.

## Supplementary Information

**Additional file 1.**

## Data Availability

The datasets generated and analyzed in the current study are available from the corresponding author on reasonable request.

## References

[1] Yamauchi A, Imagawa H, Sakakaibara K, Yokonishi H, Ueha R, Nito T, Tayama M, Yamasoba T (2014). Vocal fold atrophy in a Japanese tertiary medical institute: status quo of the most aged country. J Voice.

[2] Roy N, Stemple J, Merrill RM, Thomas L (2007). Epidemiology of voice disorders in the elderly: preliminary findings. Laryngoscope..

[3] Golub JS, Chen PH, Otto KJ, Hapner E, Johns MM (2006). Prevalence of perceived dysphonia in a geriatric population. J Am Geriatr Soc.

[4] Kendall K (2007). Presbyphonia: a review. Curr Opin Otolaryngol Head.Neck Surg..

[5] Goncalves TM, Martins RHG, Adriana BBP (2018). Transmission electron microscopy of the presbylarynx in the process of voice aging. J Voice.

[6] Mallick AS, Garas G, McGlashan J (2019). Presbylaryngis: a state-of-the-art review. Curr Opin Otolaryngol Head Neck Surg.

[7] Kwon TK, An SY, Ahn JC, Kim KH, Sung MW (2010). Calcium hydroxylapatite injection laryngoplasty for the treatment of presbylaryngis: long-term results. Laryngoscope..

[8] Allensworth JJ, O'Dell K, Ziegler A, Bryans L, Flint P, Schindler J (2019). Treatment outcomes of bilateral medialization thyroplasty for presbylaryngis. J Voice.

[9] Sauder C, Roy N, Tanner K, Houtz DR, Smith ME (2010). Vocal function exercises for presbylaryngis: a multidimensional assessment of treatment outcomes. Ann Otol Rhinol Laryngol.

[10] Hirano S, Sugiyama Y, Kaneko M, Mukudai S, Fuse S, Hashimoto K (2020). Intracordal injection of basic fibroblast growth factor in 100 cases of vocal fold atrophy and scar. Laryngoscope..

[11] Bhatt NK, Gao WZ, Timmons L, Sund CME, O'Dell K, Johns MM 3rd. Platelet-rich plasma for vocal fold scar: A preliminary report of concept. J Voice. 2021. Online ahead of print. 10.1016/j.jvoice.2020.12.040 33446439

[12] Zelenik K, Formanek M, Walderova R, Formankova D, Kominek P (2021). Five-year results of vocal fold augmentation using autologous fat or calcium hydroxylapatite. Eur Arch Otorhinolaryngol.

[13] Kim IG, Park MR, Choi YH, Choi JS, Ahn HJ, Kwon SK, Lee JH (2019). Regeneration of paralyzed vocal fold by the injection of plasmid DNA complex-loaded hydrogel bulking agent. ACS Biomater Sci Eng.

[14] Kwon SK, Song JJ, Cho CG, Park SW, Choi SJ, Oh SH, Lee JH (2013). Polycaprolactone spheres and theromosensitive Pluronic F127 hydrogel for vocal fold augmentation: in vivo animal study for the treatment of unilateral vocal fold palsy. Laryngoscope..

[15] Kwon SK, Kim HB, Song JJ, Cho CG, Park SW, Choi JS, Ryu J, Oh SH, Lee JH (2014). Vocal fold augmentation with injectable polycaprolactone microspheres/pluronic F127 hydrogel: long-term in vivo study for the treatment of glottal insufficiency. PLoS One.

[16] Lim YM, Kim BH, Kim HB, Park E, Park SW, Park JS, Choi SI, Kwon TK, Kwon SK (2015). Vocal fold augmentation with beta glucan hydrogel cross-linked by gamma irradiation for enhanced duration of effect: in vivo animal study. Biomed Res Int.

[17] Choi YH, Kim SH, Kim IG, Lee JH, Kwon SK (2019). Injectable basic fibroblast growth factor-loaded alginate/hyaluronic acid hydrogel for rejuvenation of geriatric larynx. Acta Biomater.

[18] Choi YH, Ahn HJ, Park MR, Han MJ, Lee JH, Kwon SK (2019). Dual growth factor-immobilized bioactive injection material for enhanced treatment of glottal insufficiency. Acta Biomater.

[19] Chung EJ, Choi JS, Shin J, Cho H, Kim S, Park YJ, Lee Y, Kim Y, Wu H, Cho S, Kwon SK (2020). Prevention of irradiation-induced damage to salivary glands by local delivery of adipose-derived stem cells via hyaluronic acid-based hydrogels. J Ind Eng Chem.

[20] Shin J, Lee JS, Lee C, Park HJ, Yang K, Jin Y, Ryu JH, Hong KS, Moon SH, Chung HM, Yang HS, Um SH, Oh JW, Kim DI, Lee H, Cho SW (2015). Tissue adhesive catechol-modified hyaluronic acid hydrogel for effective, minimally invasive cell therapy. Adv Funct Mater.

[21] Park HJ, Jin Y, Shin J, Yang K, Lee C, Yang HS, Cho SW (2016). Catechol-functionalized hyaluronic acid hydrogels enhance angiogenesis and osteogenesis of human adipose-derived stem cells in critical tissue defects. Biomacromolecules..

[22] Pak CS, Heo CY, Shin J, Moon SY, Cho SW, Kang HJ (2021). Effects of a catechol-functionalized hyaluronic acid patch combined with human adipose-derived stem cells in diabetic wound healing. Int J Mol Sci.

[23] Kim HS, Yang J, Kim K, Shin US (2019). Biodegradable and injectable hydrogels as an immunosuppressive drug delivery system. Mater Sci Eng C Mater Biol Appl.

[24] Liechty KW, MacKenzie TC, Shaaban AF, Radu A, Moseley AM, Deans R, Marshak DR, Flake AW (2000). Human mesenchymal stem cells engraft and demonstrate site-specific differentiation after in utero transplantation in sheep. Nat Med.

[25] Lee DY, Kim HB, Shim IJ, Kanai N, Okano T, Kwon SK (2017). Treatment of chemically induced oral ulcer using adipose-derived mesenchymal stem cell sheet. J Oral Pathol Med.

[26] Kim IG, Park SA, Lee SH, Choi JS, Cho H, Lee SJ, Kwon YW, Kwon SK (2020). Transplantation of a 3D-printed tracheal graft combined with iPS cell-derived MSCs and chondrocytes. Sci Rep.

[27] Lee DY, Lee JH, Ahn HJ, Oh SH, Kim TH, Kim HB, Park SW, Kwon SK (2015). Synergistic effect of laminin and mesenchymal stem cells on tracheal mucosal regeneration. Biomaterials..

[28] Liang Q, Liu S, Han P, Li X, Li X, Zhao Y, Liang Y, Deng Z, Jin Y (2012). Micronized acellular dermal matrix as an efficient expansion substrate and delivery vehicle of adipose-derived stem cells for vocal fold regeneration. Laryngoscope..

[29] Niibe K, Ohori-Morita Y, Zhang M, Mabuchi Y, Matsuzaki Y, Egusa H (2020). A shaking-culture method for generating bone marrow derived mesenchymal stromal/stem cell-spheroids with enhanced multipotency in vitro. Front Bioeng Biotechnol.

[30] Rodeno MT, Sanchez-Fernandez JM, Rivera-Pomar JM (1993). Histochemical and morphometrical ageing changes in human vocal cord muscles. Acta Otolaryngol.

[31] Hirano M, Kurita S, Sakaguchi S (1989). Ageing of the vibratory tissue of human vocal folds. Acta Otolaryngol.

[32] Branco A, Rodrigues SA, Fabro AT, Fonseca-Alves CE, Martins RH (2014). Hyaluronic acid behavior in the lamina propria of the larynx with advancing age. Otolaryngol Head Neck Surg.

[33] Figueroa FE, Carrion F, Villanueva S, Khoury M (2012). Mesenchymal stem cell treatment for autoimmune diseases: a critical review. Biol Res.

[34] Djouad F, Bouffi C, Ghannam S, Noel D, Jorgensen C (2009). Mesenchymal stem cells: innovative therapeutic tools for rheumatic diseases. Nat Rev Rheumatol.

[35] Yeh HY, Liu BH, Sieber M, Hsu SH (2014). Substrate-dependent gene regulation of self-assembled human MSC spheroids on chitosan membranes. BMC Genomics.

[36] Cheng NC, Chen SY, Li JR, Young TH (2013). Short-term spheroid formation enhances the regenerative capacity of adipose-derived stem cells by promoting stemness, angiogenesis, and chemotaxis. Stem Cells Transl Med.

[37] Cesarz Z, Funnell JL, Guan J, Tamama K (2016). Soft elasticity-associated signaling and bone morphogenic protein 2 are key regulators of mesenchymal stem cell spheroidal aggregates. Stem Cells Dev.

[38] Bartosh TJ, Ylöstalo JH, Mohammadipoor A, Bazhanov N, Coble K, Claypool K, Lee RH, Choi H, Prockop DJ (2010). Aggregation of human mesenchymal stromal cells (MSCs) into 3D spheroids enhances their antiinflammatory properties. Proc Natl Acad Sci U S A.

[39] Lee JH, Han YS, Lee SH (2016). Long-duration three-dimensional spheroid culture promotes angiogenic activities of adipose-derived mesenchymal stem cells. Biomol Ther (Seoul).

[40] Oberringer M, Bubel M, Jennewein M, Guthörl S, Morsch T, Bachmann S, Metzger W, Pohlemann T (2018). The role of adipose-derived stem cells in a self-organizing 3D model with regard to human soft tissue healing. Mol Cell Biochem.

[41] Cesarz Z, Tamama K (2016). Spheroid culture of mesenchymal stem cells. Stem Cells Int.

[42] Li Y, Guo G, Li L, Chen F, Bao J, Shi YJ, Bu H (2015). Three-dimensional spheroid culture of human umbilical cord mesenchymal stem cells promotes cell yield and stemness maintenance. Cell Tissue Res.

[43] Mann CJ, Perdiguero E, Kharraz Y, Aguilar S, Pessina P, Serrano AL, Muñoz-Cánoves P (2011). Aberrant repair and fibrosis development in skeletal muscle. Skelet Muscle.

[44] Duan Y, Liu Z, O'Neill J, Wan LQ, Freytes DO, Vunjak-Novakovic G (2011). Hybrid gel composed of native heart matrix and collagen induces cardiac differentiation of human embryonic stem cells without supplemental growth factors. J Cardiovasc Transl Res.

[45] Sellaro TL, Ranade A, Faulk DM, McCabe GP, Dorko K, Badylak SF, Strom SC (2010). Maintenance of human hepatocyte function in vitro by liver-derived extracellular matrix gels. Tissue Eng Part A.

[46] O'Neill JD, Freytes DO, Anandappa AJ, Oliver JA, Vunjak-Novakovic GV (2013). The regulation of growth and metabolism of kidney stem cells with regional specificity using extracellular matrix derived from kidney. Biomaterials..

[47] Lungova V, Leydon C, Thibeault S (2016). Derivation of epithelial cells from human embryonic stem cells as an in vitro model of vocal mucosa. Methods Mol Biol.

[48] Peng H, Ming L, Yang R, Liu Y, Liang Y, Zhao Y, Jin Y, Deng Z (2013). The use of laryngeal mucosa mesenchymal stem cells for the repair the vocal fold injury. Biomaterials..

[49] Bless DM, Hirano M, Feder RJ (1987). Videostroboscopic evaluation of the larynx. Ear Nose Throat J.

