# Supplementary material for: Injection laryngoplasty of human adipose-derived stem cell spheroids with hyaluronic acid-based hydrogel improves the morphological and functional characteristics of geriatric larynx
Source: Biomater Res. 2022 Apr 5;26:13. doi: 10.1186/s40824-022-00261-x (PMC8981753; doi:10.1186/s40824-022-00261-x)
Supplement: Supplementary file 1 — Additional file 1. [file 40824_2022_261_MOESM1_ESM.zip › Supplementary Data.docx]

**Supplementary Data**


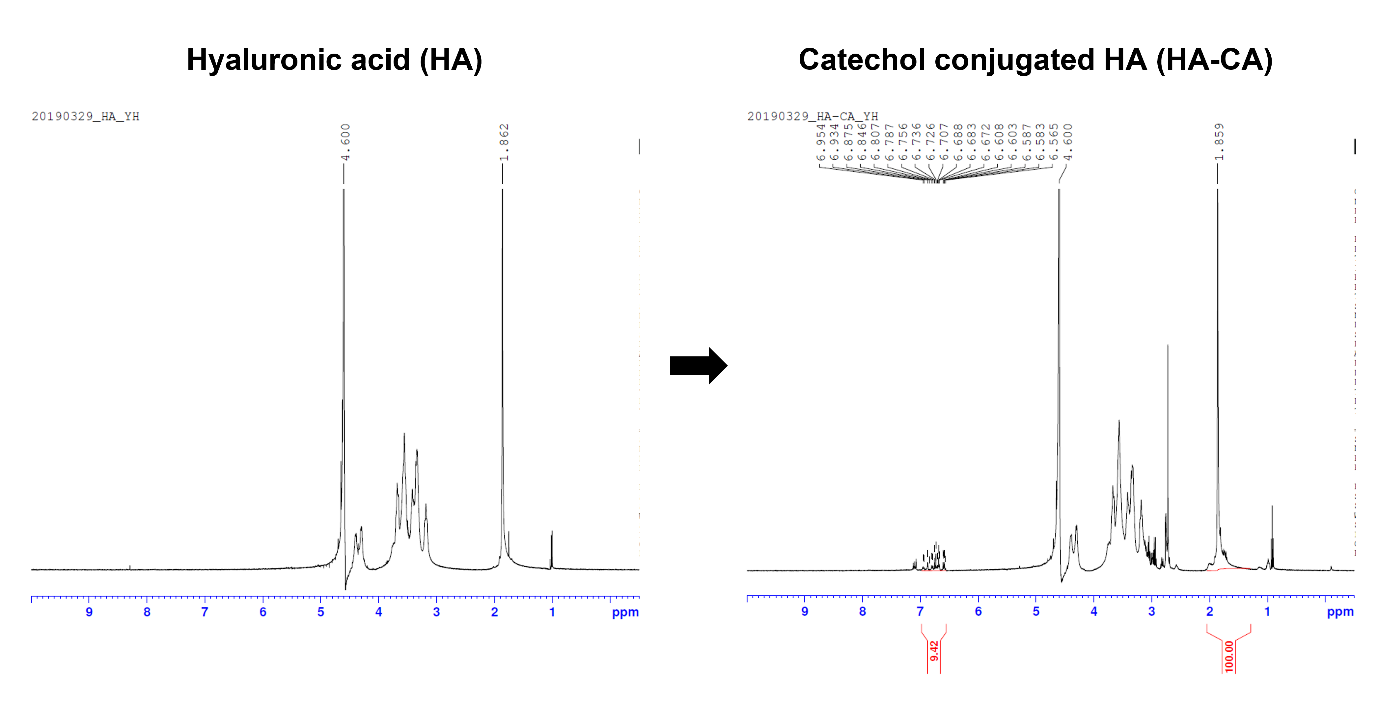


**Supplementary Figure 1. ^1^H NMR** **analysis of catechol-conjugated hyaluronic acid (HA-CA)**

The degree of catechol conjugation (9.42 % shown) was determined by comparison of the peak integrals at 7.8-7.2 ppm and 1.8-2.2 ppm


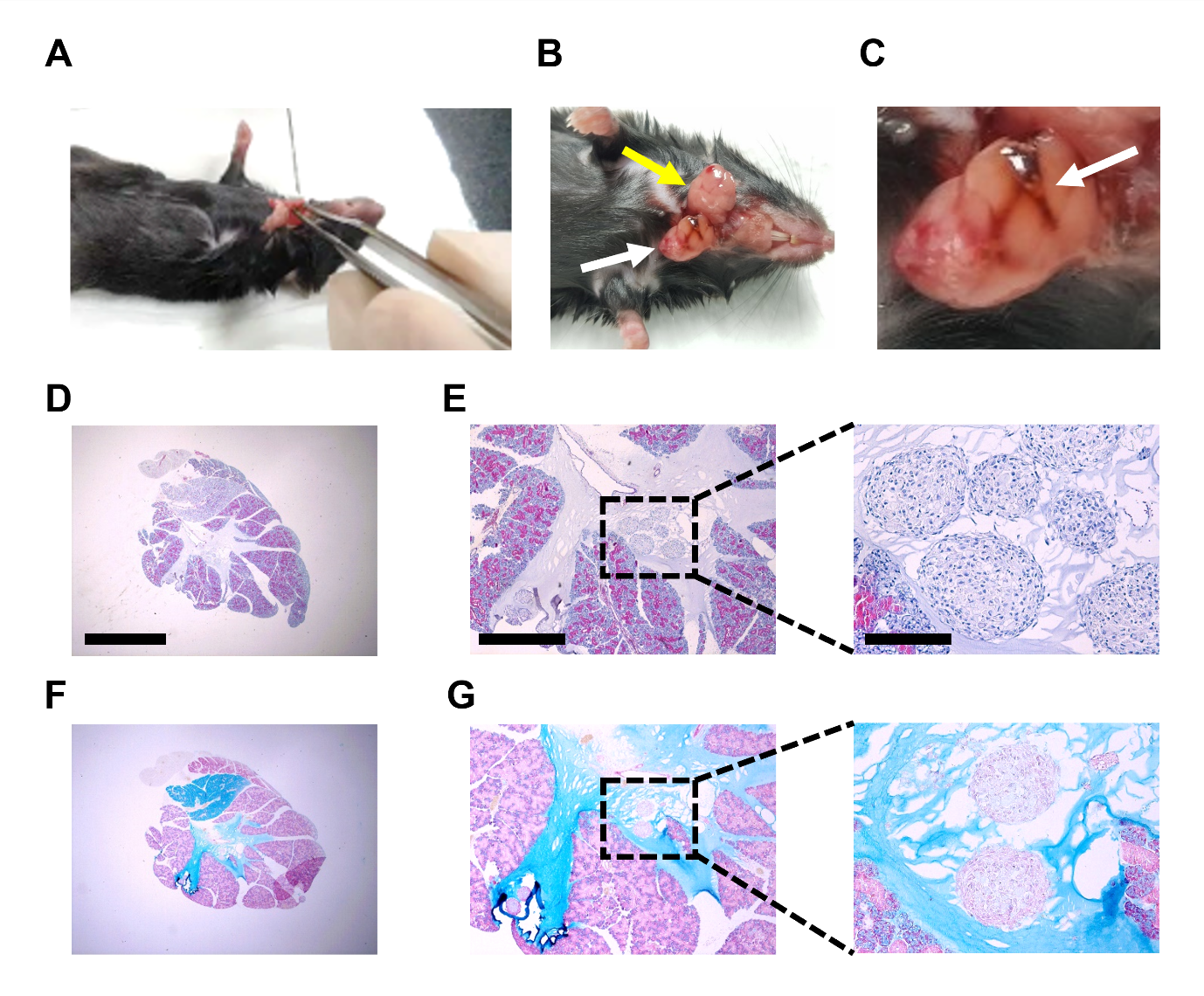


**Supplementary Figure 2. Injection of HA-CA hydrogel carrying hASC spheroids into the mouse salivary gland.**

(A) HA-CA hydrogel carrying hASC spheroids was injected into the mouse salivary gland with a 27-gauge spinal needle mounted with Hamilton syringe. (B) Hydrogel injected salivary gland (white arrow) and no-treated salivary gland (yellow arrow). (C) hASC Spheroids were well localized in the salivary gland with hydrogel (white arrow). (D and E) Haematoxylin and eosin staining of salivary gland 3 days after injection (scale bar of D = 3 mm, scale bar of E left = 1 mm, and scale bar of E right = 200 µm). (F and G) Alcian blue staining of salivary gland 3 days after injection.
