# Supplementary figures and images for: Injection laryngoplasty of human adipose-derived stem cell spheroids with hyaluronic acid-based hydrogel improves the morphological and functional characteristics of geriatric larynx
Source: Biomater Res. 2022 Apr 5;26:13. doi: 10.1186/s40824-022-00261-x (PMC8981753; doi:10.1186/s40824-022-00261-x)

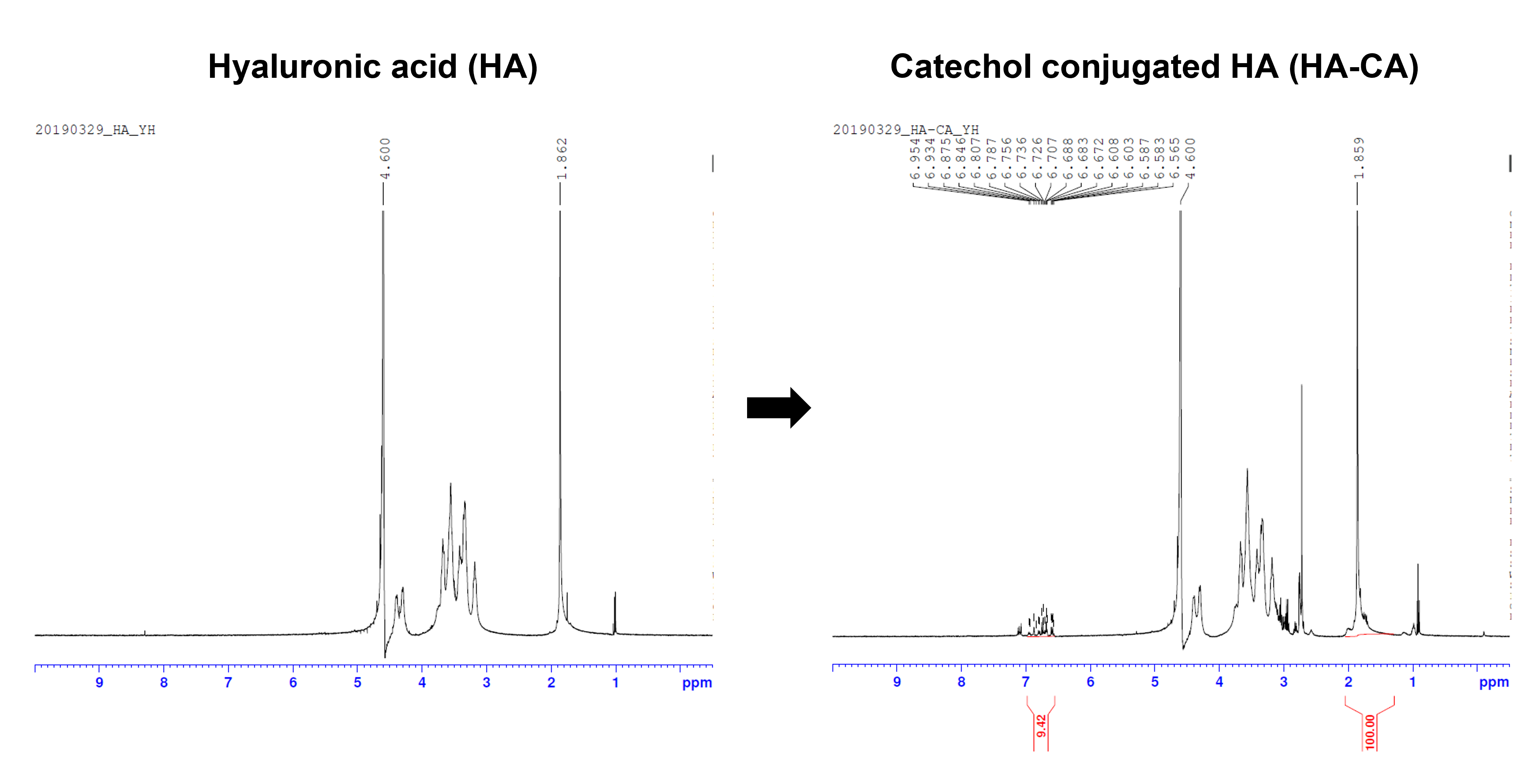

Supplement: Supplementary file 1 — Additional file 1. [file 40824_2022_261_MOESM1_ESM.zip › Supplementary figure 1.png]
